# Genetic Identification of F1 and Post-F1 Serrasalmid Juvenile Hybrids in Brazilian Aquaculture

**DOI:** 10.1371/journal.pone.0089902

**Published:** 2014-03-03

**Authors:** Diogo Teruo Hashimoto, José Augusto Senhorini, Fausto Foresti, Paulino Martínez, Fábio Porto-Foresti

**Affiliations:** 1 Centro de Aquicultura, Universidade Estadual Paulista, (UNESP), Campus de Jaboticabal, Jaboticabal, SP, Brazil; 2 Centro de Pesquisa e Gestão de Recursos Pesqueiros Continentais, Instituto Chico Mendes de Conservação da Biodiversidade, Pirassununga, SP, Brazil; 3 Departamento de Morfologia, Instituto de Biociências, Universidade Estadual Paulista, (UNESP), Campus de Botucatu, Botucatu, SP, Brazil; 4 Departamento de Genética, Universidad de Santiago de Compostela, Facultad de Veterinaria, Lugo, Spain; 5 Departamento de Ciências Biológicas, Faculdade de Ciências, Universidade Estadual Paulista, (UNESP), Campus de Bauru, Bauru, SP, Brazil; Wageningen UR Livestock Research, Netherlands

## Abstract

Juvenile fish trade monitoring is an important task on Brazilian fish farms. However, the identification of juvenile fish through morphological analysis is not feasible, particularly between interspecific hybrids and pure species individuals, making the monitoring of these individuals difficult. Hybrids can be erroneously identified as pure species in breeding facilities, which might reduce production on farms and negatively affect native populations due to escapes or stocking practices. In the present study, we used a multi-approach analysis (molecular and cytogenetic markers) to identify juveniles of three serrasalmid species (*Colossoma macropomum*, *Piaractus mesopotamicus* and *Piaractus brachypomus*) and their hybrids in different stocks purchased from three seed producers in Brazil. The main findings of this study were the detection of intergenus backcrossing between the hybrid ♀ patinga (*P. mesopotamicus*×*P. brachypomus*)×♂ *C. macropomum* and the occurrence of one hybrid triploid individual. This atypical specimen might result from automixis, a mechanism that produces unreduced gametes in some organisms. Moreover, molecular identification indicated that hybrid individuals are traded as pure species or other types of interspecific hybrids, particularly post-F1 individuals. These results show that serrasalmid fish genomes exhibit high genetic heterogeneity, and multi-approach methods and regulators could improve the surveillance of the production and trade of fish species and their hybrids, thereby facilitating the sustainable development of fish farming.

## Introduction

In Brazil, approximately 40 native fish species and 6 interspecific hybrids are cultivated on fish farms [1], [2]. The representatives of the Serrasalmidae family, *i.e.*, *Colossoma macropomum* (tambaqui), *Piaractus mesopotamicus* (pacu), *Piaractus brachypomus* (pirapitinga or caranha), and their interspecific hybrids tambacu (female tambaqui×male pacu), patinga (female pacu×male pirapitinga), and tambatinga (female tambaqui×male pirapitinga) correspond to native fish with the largest production in Brazilian aquaculture (56.2 million kg per year) according to IBAMA (Instituto Brasileiro do Meio Ambiente e dos Recursos Naturais Renováveis) [3]. Reciprocal hybrids (*e.g.*, female pacu x male tambaqui) are also viable [4], but these individuals are not typically produced or cultivated on fish farms.

The cultivation of serrasalmids varies among different regions of Brazil [2]. In Southern Brazil, the only species produced is pacu. Tambaqui and pirapitinga, and their hybrids, are not produced in this region because these species cannot tolerate the low temperatures of Southern Brazil. In Northern Brazil, serrasalmid hybrids are produced at a lower rate compared with the pure species tambaqui, the main aquaculture resource in this region. In contrast, hybrids are associated with high production rates in the other regions of Brazil, particularly in the Midwest [3], and the hybrid tambacu has a greater economic importance than other serrasalmid hybrids. This fish group is also widely farmed in other Latin American (Colombia, Venezuela, and Cuba) [5] and Asian (China, Myanmar, Thailand, and Vietnam) countries [6], [7].

However, the diversity and zootechnical differences among fish are problematic for the aquaculture industry because pure species or their hybrids can be produced or cultivated as a single species. This inaccuracy primarily reflects the morphological similarity between species, particularly in the case of hybrids and parental species in the juvenile stage. Thus, the use of genetic markers is essential to monitor the production and management of fish hybrid, particularly for the trade between seed suppliers and fish farmers, which is a critical point in the production chain [8].

Currently, PCR-RFLP (polymerase chain reaction - restriction fragment length polymorphism) and multiplex-PCR have been characterized as efficient methods for the rapid and inexpensive identification of hybrids [9]–[11]. For serrasalmid hybrids, these methodologies facilitate diagnoses based on the combination of single nucleotide variants in the mitochondrial genes, Cytochrome C Oxidase subunit I (*mt-co1*) and Cytochrome b (*mt-cyb*), with nuclear genes, such as α-Tropomyosin (*tpm1*) and Recombination Activating Gene 2 (*rag2*) [12]. Nuclear diagnostic markers are essential to differentiate hybrids between species, but mitochondrial markers, although haploid, identify the maternal origin of hybrids, and this information is crucial for the assessment of hybridization.

Cytogenetic analysis methods have also been described for the identification of serrasalmid hybrids. Through C-banding and fluorescence *in situ* hybridization (18S ribosomal RNA probe), chromosome markers have facilitated the precise identification of the hybrids tambacu and tambatinga, respectively [13], [14]. Although cytogenetic methods have limitations of low throughput because of the effort and time required for data analysis and processing [2], these techniques provide important information to verify ploidy level [13], which cannot be directly assessed through molecular markers.

Despite hybrid vigor in some cases, there are problems associated with the inadequate use of interspecific hybrids for aquaculture production. Occasionally, fish farmers have mistakenly used hybrids as broodstock, as reported for tilapia, catfish, and carp [15]–[17]. Superior performance or desirable characteristics associated with hybrid vigor might be lost in post-F1 individuals because introgressive hybridization reduces the heterosis obtained in F1 hybrids, and particularly because post-F1 hybrids typically show reduced offspring viability due to high mortality rates [18]. These observations have been previously reported in catfish, where hybrids were used as broodstock [17].

In the present study, we used PCR-RFLP, multiplex-PCR, and cytogenetic methods to evaluate the juvenile fish trade between seed suppliers and fish farmers in Brazil, focusing on the genetic identification of F1 and post-F1 serrasalmid hybrids. The novelty of this study was the discovery of fertility in the hybrid patinga (female pacu x male pirapitinga), and its use as broodstock in Brazilian aquaculture. Moreover, mistaken trade of hybrid tambacu was detected in fish farms and one post-F1 hybrid was characterized as triploid.

## Materials and Methods

We performed the genetic identification of 924 juvenile individuals from eight stocks of live fish purchased from three private Brazilian aquaculture seed producers (herein referred to as SPS, MGS, and SES) (Table 1). All fish farms assessed in this study represent large companies in Brazil. From fish farm SPS, located in São Paulo State (Southeastern Brazil), we analyzed two commercially available stocks, labeled as hybrids tambacu (SPS1) and patinga (SPS2). From fish farm MGS, located in the Minas Gerais State (Southeastern Brazil), we analyzed three commercially produced stocks, labeled as pure tambaqui (MGS1), pacu (MGS2), and hybrid tambacu (MG3). From fish farm SES, located in Sergipe State (Northeastern Brazil), we analyzed three commercially stocks, labeled as pure tambaqui (SES1), hybrids tambatinga (SES2), and tambacu (SES3). The size of the analyzed fish ranged from 5 to 10 cm. We did not notify the producers that the fish would be used for identification purposes.

**Table 1:** Molecular identification of juvenile serrasalmid fish stocks purchased from different fish farmers.

Fish farm	Stocks purchased	Stock identification	n
SPS (São Paulo State)	tambacu	SPS1	50
	patinga	SPS2	33
MGS (Minas Gerais State)	tambaqui	MGS1	143
	pacu	MGS2	115
	tambacu	MGS3	133
SES (Sergipe State)	tambaqui	SES1	150
	tambatinga	SES2	150
	tambacu	SES3	150

This study was conducted in strict accordance with the recommendations of the National Council for Control of Animal Experimentation (Brazilian Ministry for Science, Technology and Innovation). The present study was performed under authorization N° 33435-1 issued through ICMBio (Chico Mendes Institute for the Conservation of Biodiversity, Brazilian Ministry for Environment). Fin fragments were collected from each fish under benzocaine anesthesia and all efforts were made to minimize suffering. DNA was extracted from the fin fragments using the Wizard Genomic DNA Purification Kit (Promega) according the manufacturer's protocol. The DNA concentration was assessed against a molecular marker standard (the Low DNA Mass Ladder, Invitrogen) through electrophoresis on a 1% agarose gel.

The samples were genotyped using two methods and different genes, as previously described [12]: 1) multiplex-PCR based on nuclear α-Tropomyosin (*tpm1*) and mitochondrial Cytochrome C Oxidase subunit I (*mt-co1*) genes; and 2) PCR-RFLP using the nuclear Recombination Activating Gene 2 (*rag2*) and mitochondrial Cytochrome b (*mt-cyb*) genes. Both methods provide diagnostic electrophoretic fragments for each parental species and their interspecific hybrids. Diagnostic sizes of the PCR products or restriction fragments are described in the Table 2. The sequences for the primers and restriction enzymes, PCR reagents, reagent concentrations, and reaction conditions were used as previously described [12]. We used multiplex-PCR, followed by PCR-RFLP in subsequent analyses for confirmation. DNA samples from the pure parental species were used as controls for reaction specificity in all experiments. These samples were previously identified through morphological and molecular analyses [12] and obtained from the stock maintained at the Centro Nacional de Pesquisa e Conservação de Peixes Continentais (CEPTA/ICMBio, Pirassununga, São Paulo State, Brazil).

**Table 2:** Sizes of the PCR products or restriction fragments, according to Hashimoto et al. [12].

		Diagnostic fragment size (bp)		
Method	Gene	pacu	tambaqui	pirapitinga
Multiplex-PCR	*mt-co1*	307	435	307 and 610
	*tpm1*	269	172	131
PCR-RFLP	*mt-cyb*	152 and 513	261 and 405	665
	*rag2*	750	357 and 393	250 and 500

Cytogenetic analysis was also performed to verify the ploidy level in individuals of the SPS2 stock. Chromosomal preparations were obtained according to the methods of Foresti et al. [19]. The chromosome morphology was determined based on the arm ratio consistent with Levan et al. [20], and the chromosomes were subsequently classified as metacentric (m), submetacentric (sm), subtelocentric (st), and acrocentric (a). Fluorescence *in situ* hybridization (FISH) was performed using the method of Yang et al. [21]. These experiments encompassed all of the genotypes shown below through the molecular identification of the SPS2 stock. The 5S ribosomal RNA (rRNA) gene sequences were PCR amplified from DNA using the primers described by Pendás et al. [22]. To prepare the probe, PCR products of the 5S rRNA gene were labeled with biotin-16-dUTP (Roche) through nick translation (Invitrogen). The chromosomes were counterstained with DAPI (4′,6-diamidino-2-phenylindole, Vector Laboratories). The FISH images were captured and processed using the CytoVision Genus system (Applied Imaging, USA) and a Cohu CCD camera mounted on an Olympus BX-60 microscope.

## Results

**Figure 1:**
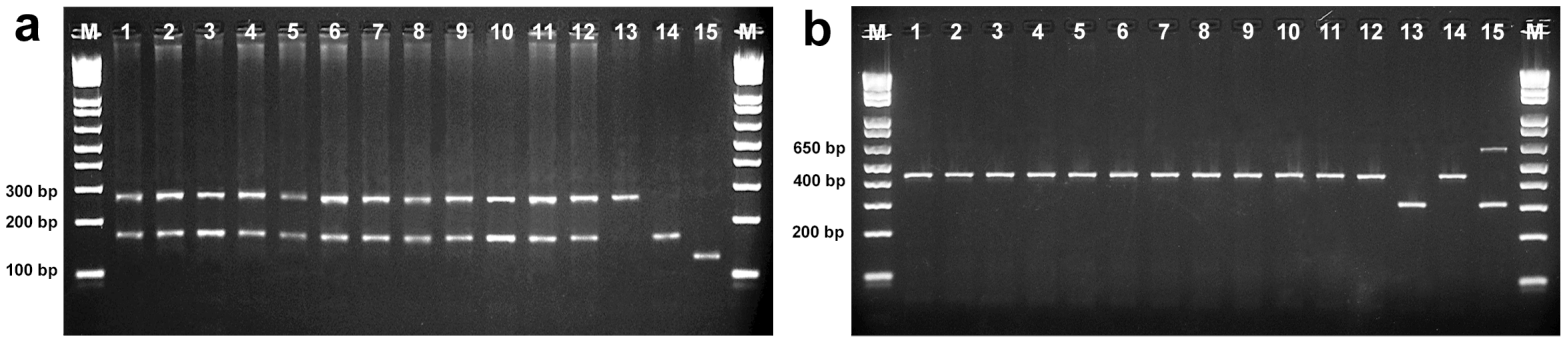
Molecular identification of the samples SPS1, MGS1, MGS2, MGS3, SES1, SES2, and SES3 using the nuclear *tpm1* (a) and mitochondrial *mt-co1* (b) genes in multiplex-PCR. Lanes 1–12, genotypes of hybrid tambacu (♀ tambaqui x ♂ pacu); and lanes 13, 14, and 15, control DNA samples from the pure pacu, tambaqui, and pirapitinga species, respectively; M, 1 Kb Plus DNA Ladder.

We obtained the same genotype with all molecular markers in the samples purchased as hybrid tambacu (stocks SPS1, MGS3, and SES3), pure tambaqui (MGS1 and SES1), pacu (MGS2), and hybrid tambatinga (SES2), indicating that these species correspond to hybrid tambacu. The results of the multiplex-PCR analysis of the nuclear marker *tpm1* (Figure 1a) revealed a heterozygous genotype (fragments of 172 and 269 bp), characteristic of hybrid tambacu. Moreover, multiplex-PCR of the mitochondrial marker *mt-co1* (Figure 1b) showed that these hybrids exhibited the genotype of the maternal species tambaqui (fragment of 435 bp), consistent with the identification of these samples as tambacu (♀ tambaqui x ♂ pacu) instead of the reciprocal hybrid paqui (♀ pacu×♂ tambaqui). The results were confirmed through PCR-RFLP using the nuclear *rag2* and mitochondrial *mt-cyb* genes. Thus, the MGS1, MGS2, SES1, and SES2 samples were mislabeled, as these species were actually tambacu.

**Figure 2:**
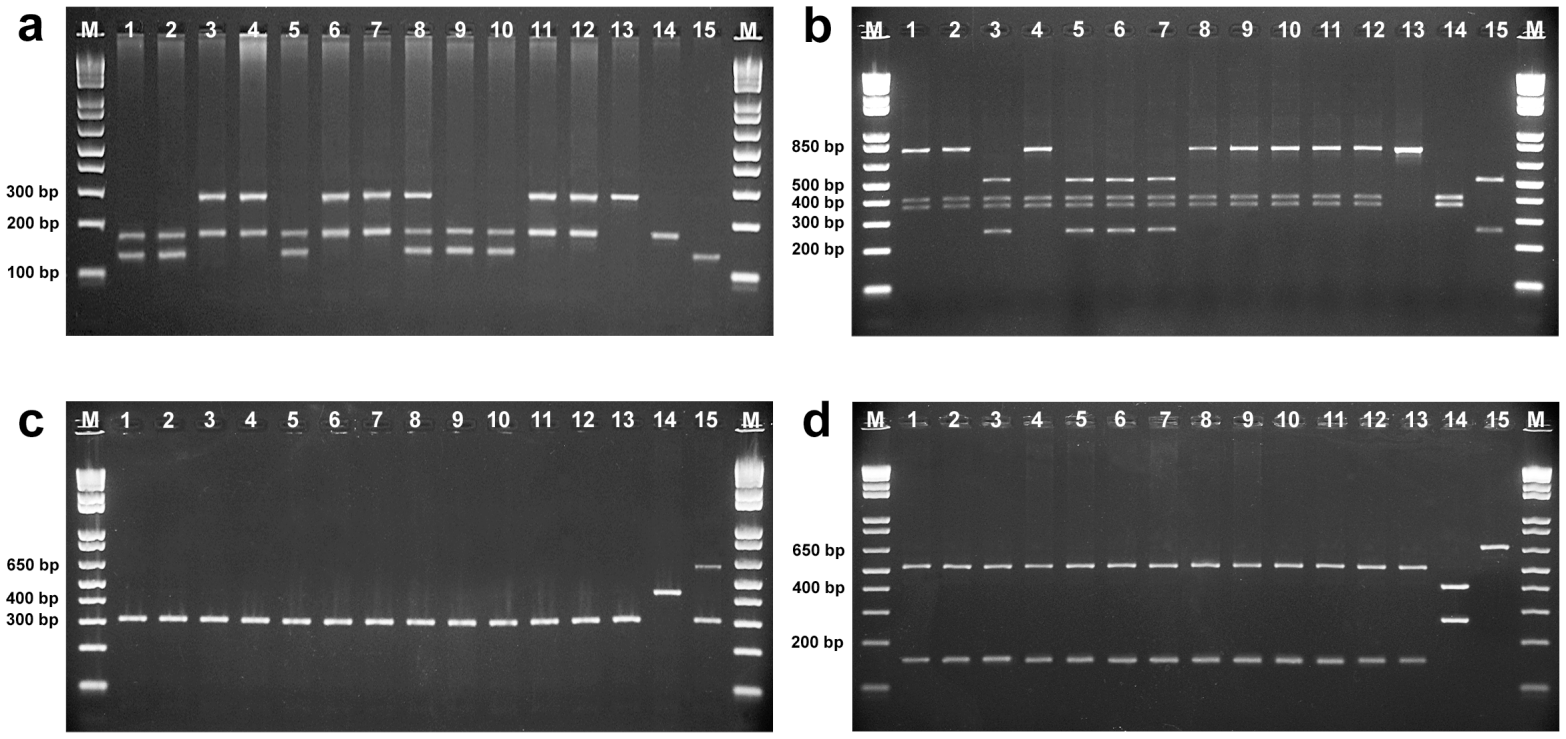
Molecular identification of the SPS2 stock using the nuclear *tpm1* (a) and *rag2* (b) genes, and mitochondrial *mt-co1* (c) and *mt-cyb* (d) genes. Lanes 4, 11, and 12, genotype A; lane 5, genotype B; lanes 3, 6, and 7, genotype C; lanes 1, 2, 9, and 10, genotype D; lane 8, genotype E; and lanes 13, 14, and 15, control DNA samples from the pure pacu, tambaqui, and pirapitinga species, respectively; M, 1 Kb Plus DNA Ladder.

**Figure 3:**
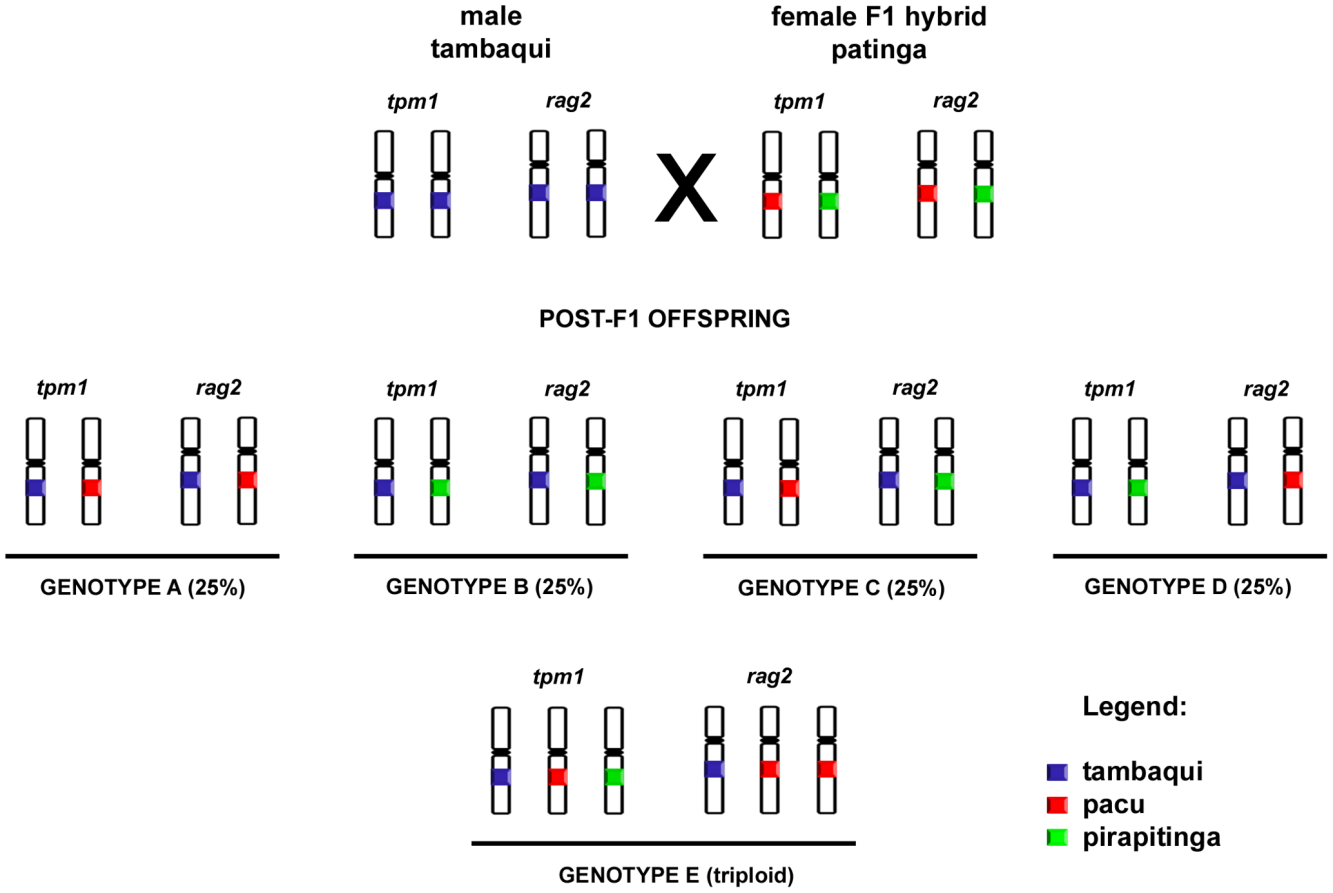
Schematic representation of the backcrossing between ♂ tambaqui x ♀ patinga (♀ pacu x ♂ pirapitinga), demonstrating the *tpm1* and *rag2* gene loci and the expected probability of each genotype.

The results of molecular identification in the SPS2 stock demonstrated that these juveniles likely correspond to post-F1 hybrids, resulting from the backcrossing of the hybrid ♀ patinga (♀ pacu x ♂ pirapitinga) with ♂ tambaqui. Consistent with this hypothesis, all the offspring showed the pacu genotypes for the mitochondrial *mt-co1* (fragment of 307 bp) and *mt-cyb* (fragments of 152 and 513 bp) genes, and segregating genotypes at the nuclear *tpm1* and *rag2* markers, as indicated below (Figures 2 and 3):

Genotype A (8 individuals): pattern of hybrid tambacu for both nuclear markers *tpm1* (fragments of 172 and 269 bp) and *rag2* (fragments of 357, 393, and 750 bp).
Genotype B (9 individuals): pattern of hybrid tambatinga for both nuclear markers *tpm1* (131 and 172 bp) and *rag2* (250, 357, 393, and 500 bp).
Genotype C (6 individuals): nuclear markers of hybrid tambacu for the *tpm1* (172 and 269 bp) gene, and genotype of hybrid tambatinga for the *rag2* (250, 357, 393, and 500 bp) gene.
Genotype D (9 individuals): opposite nuclear patterns to the genotype C, *i.e.*, genotype of hybrid tambatinga for the *tpm1* (131 and 172 bp) gene, and genotype of hybrid tambacu for the *rag2* (357, 393, and 750 bp) gene.
Genotype E (1 individual): atypical genotype comprising gene fragments from the three pure species (tambaqui, pacu, and pirapitinga) for the *tpm1* (131, 172, and 269 bp) gene, and genotype of hybrid tambacu for the *rag2* (357, 393, and 750 bp) gene. This unexpected pattern is compatible with a triploid individual, consistent with the cytogenetic results shown below.

According to the Mendelian inheritance, the hypothesized backcross ♀ patinga (♀ pacu x ♂ pirapitinga) x ♂ tambaqui would produce each A–D genotype at 25% among the offspring (Figure 3), and the observed data did not deviate from the null hypothesis, confirmed using the χ2 test (p = 0.86).

**Figure 4:**
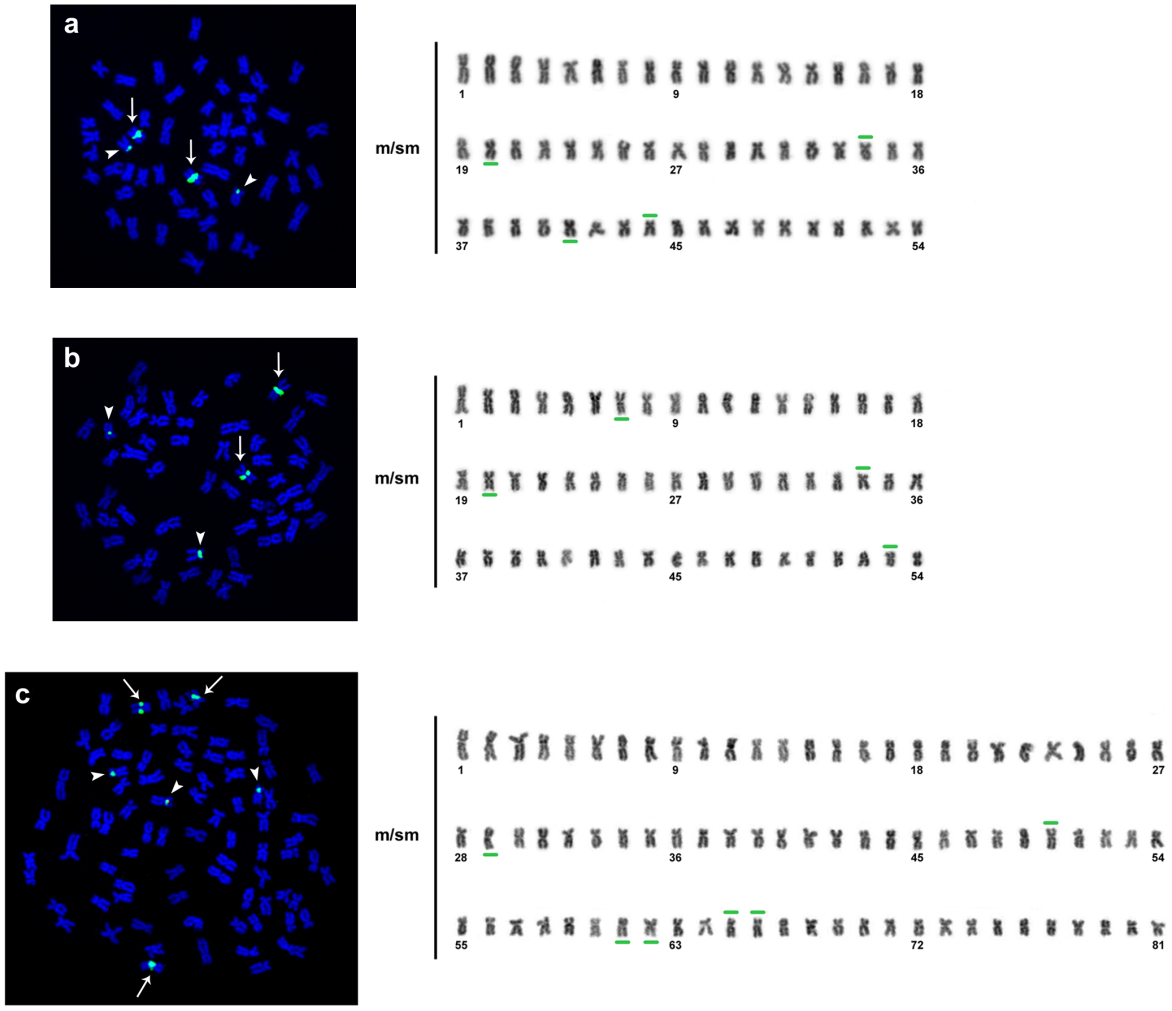
Metaphases and the respective karyotypes of individuals of the SPS2 stock, showing the chromosome location of the 5S rRNA clusters. The arrows and arrowheads indicate the 5S rRNA major and minor clusters, respectively, in metaphase. In the karyotypes, the green bars indicate the chromosomes bearing 5S rRNA genes and their respective locations (long or short arms). (a) an individual of genotype A, (b) an individual of genotype B, and (c) an individual of genotype E.

**Figure 5:**
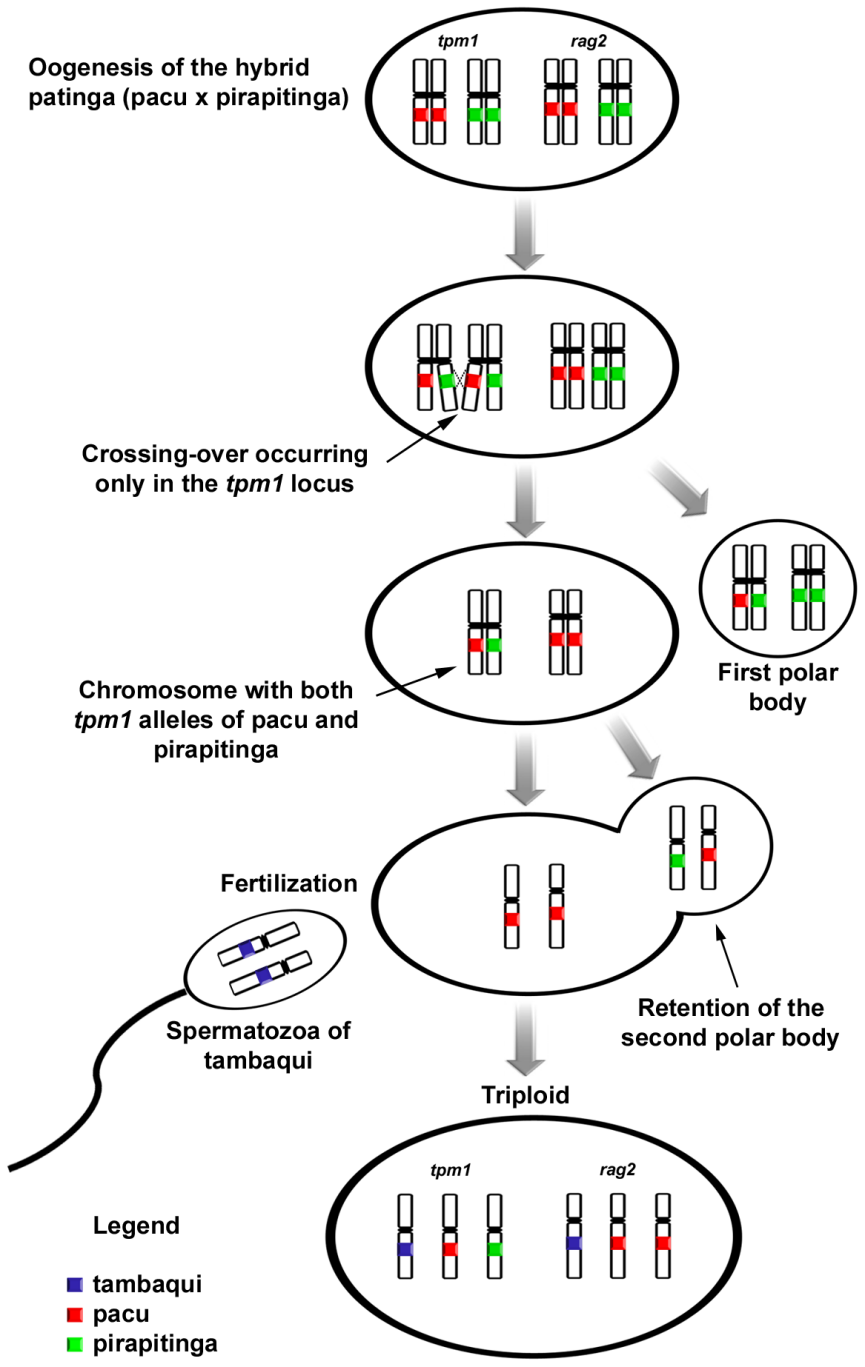
Schematic representation of the events of crossing-over and the fusion/retention of the second polar body, which likely generated the triploid individual of genotype E.

The cytogenetic analysis showed a diploid chromosome number of 2n = 54 for most individuals of the SPS2 stock, with chromosomes presenting morphologies of the types m and sm, excluding one specimen comprising 81 chromosomes (50 metaphases with this chromosome number were counted), suggesting a polyploid individual with three chromosome sets (3n) (Figure 4), and corresponding to genotype E. The occurrence of this individual might reflect a likely meiotic segregation pattern, including a crossing-over between the *tpm1* gene locus and the centromere, but not in the *rag2*-bearing chromosomes, during female oogenesis, with the fusion/retention of the second polar body in the egg, resulting in a triploid individual (Figure 5).

The FISH analysis of the specimens in the SPS2 stock revealed 5S rRNA clusters in the subcentromeric region of four chromosomes, some of which were non-homologous, as revealed through differences in morphology/size and the positions of the hybridization signals (Figures 4a and 4b). In two chromosomes, the genes were located on the long arms (major clusters), and in the other two chromosomes, the genes were located on the short arms (minor clusters). We observed different combinations of these chromosomes in the analyzed individuals, further suggesting the occurrence of post-F1 individuals. Moreover, the individual previously identified as triploid was characterized with six 5S rRNA clusters: three chromosomes with FISH signals in the subcentromeric region of the long arms (major clusters), and the other three chromosomes with signals in the short arms (minor clusters) (Figure 4c). No correlation was observed between the different chromosomes carrying 5S rRNA clusters with the genotypes of the nuclear markers *tpm1* and *rag2*, thus suggesting independent segregation between these markers.

## Discussion

Hybrids between tambaqui and pacu are popular in Brazil, as these hybrids combine the robustness and faster growth rate of tambaqui with the low temperature resistance of pacu [23], [24]. The technology required for the reproduction of hybrid tambacu through hormonal induction has been well established and widely used in farming systems, making cultivation easier than with other hybrids and even pure species. This effect might explain the results obtained in the present study, in which the hybrid tambacu was traded as other hybrids and pure species. The admixture of different types of fish is another problem observed on fish farms [12], where stocks of serrasalmid juvenile fish comprised up to five types of fish, including pure species and hybrid individuals. However, these mislabeling activities represent a fraud to the market and are not productive for cultivation and aquaculture because different hybrids and pure species have specific zootechnical characteristics and economic values [2].

Special attention should be given when hybrids are sold as pure species, as demonstrated in the present study for the stocks of tambaqui (MGS1 and SES1) and pacu (MGS2). The results showed that in addition to fraud, fish farmers are not aware of the potential biological and environmental risks represented by hybrids, whose impact could affect the aquaculture industry and threaten native species, as previously described in other species, such as tilapia, catfish, and trout [25]–[28]. Moreover, the same problems have been observed for other Brazilian fish farms in several States (São Paulo, Minas Gerais, Piauí, Sergipe, and Pará) [12], [29], indicating that could be a common practice in the aquaculture industry.

In addition, the results of the molecular characterization in the present study provided clear evidence that individuals of female patinga are fertile, similar to the hybrids tambacu and tambatinga [18], [30]. Hence, due to the difficulty of morphological identification, these hybrids can be erroneously used as broodstock on Brazilian fish farms, which is not productive for aquaculture and represents even higher risks [15], [31]. The negative effects of post-F1 individuals include the dilution or loss of the desirable characteristics, resulting from hybrid vigor [32], low hatching rate and high mortality level [17], [18]. Therefore, the data obtained in the present study show that molecular tools should be applied in breeding facilities to ensure the integrity of pure stocks used on the Brazilian fish farms, as previously demonstrated [18], where even post-F1 hybrids have been observed in the farmed broodstock of catfish.

Serrasalmid hybrids have also been detected in the natural environment and some authors have suggested that these individuals are the consequence of aquaculture activities [12], [33], [34]. The presence of fertile hybrids on the farms observed in the present study reveals the potential risks of these farming practices to wild populations. Introgressive hybridization poses a threat to the genetic integrity of pure species, which might result in a single hybrid population, as reported in trout, catfish, and tilapia [27], [35]–[37]. In Brazil, this situation has also been described for hybrids between the Neotropical catfish species *Pseudoplatystoma corruscans* and *Pseudoplatystoma reticulatum* [38]. Genetic analysis of these species have revealed a high frequency of hybrids, including post-F1 generation individuals in the Mogi Guaçu (50%) and Aquidauana (30%) rivers, where the majority of the Brazilian fish farms are located, suggesting that hybrids might be introduced from farmed stocks [38]. The results obtained from the present study should be complemented with the analysis of wild populations to confirm whether hybrids in natural environments result from farm escapees or natural hybridization. This point is critical for the development of government regulations to achieve sustainable and environmentally respected aquaculture.

The combination of molecular markers and cytogenetic techniques was essential for the identification of a triploid individual in the present study and to confirm the hybridization processes. Cytogenetic information is considered a suitable tool to verify ploidy level and analyze parental chromosome sets in hybrids [4], [39], [40]. In previous studies, pure species of tambaqui, pacu, and pirapitinga were characterized by a diploid chromosome number of 54 m/sm chromosomes [13], [14]. The individuals cytogenetically analyzed in this study also showed 2n = 54 chromosomes, with the exception of the sample corresponding to genotype E, which was characterized by triploidy (3n). This type of event was also described for an individual of the hybrid tambacu [13] and for a post-F1 specimen resulting from backcrossing between ♀ pacu x ♂ tambacu (♀ tambaqui x ♂ pacu), which also generated gynogenetic individuals [18].

Spontaneous triploid fish can be explained by several cytological mechanisms that produce unreduced diploid gametes [41]. Premeiotic endomitosis is characterized by genome doubling without cytokinesis before meiotic division, followed by two quasinormal divisions. In apomixis, meiosis is repressed in the first division, and the oocyte is produced through mitosis, without the recombination and segregation of the homologous chromosomes. However, in both mechanisms, the unreduced gametes are isogenic, *i.e.*, genetically identical to the parent without genetic variation of the resulting eggs [41], [42]. Premeiotic endomitosis and apomixis occurs in several fish species [43], [44], including interspecific hybrids [45], but cannot explain the triploid event observed in the present study, as genetic variations were detected in the molecular analyses.

Alternatively, these results are consistent with a cytological mechanism similar to automixis, in which meiosis is maintained and diploid gametes are generated after meiosis through the fusion/retention of the polar body. This phenomenon has been well documented in the triploid offspring of poeciliid interspecific hybrids [42]. The resulting triploid products are not genetically identical to the parental genome, as segregation and recombination result in nonidentical homologous chromosomes [42], explaining the atypical genotype E observed herein. However, further studies are needed to evaluate whether this triploid event is due to automixis or whether hybridization facilitates polyploidization, as shown in other species [46].

Consistent with the data obtained in this study, the presence of 5S rRNA clusters in two pairs of chromosomes in diploid individuals is a common characteristic in fish genomes [47], [48]. However, the results of the present study demonstrated the occurrence of different combinations of chromosomes bearing 5S rRNA sites, suggesting these combinations were inherited from chromosomes of distinct species.

The molecular and cytogenetic data obtained herein are consistent with the hypothesis of the fertility of hybrid patinga. However, the presence of additional post-F1 hybrids (F2 or advanced backcrosses) cannot be ruled out at least for some specimens because of the small number of nuclear markers used in this study. According to Boecklen and Horward [49], more than 70 nuclear markers are needed for the reliable differentiation between pure species and advanced hybrid crosses or backcrosses. Thus, the acquisition of additional markers based on nuclear genes is necessary for the identification of serrasalmid hybrids. The combination of next-generation sequencing technologies with restriction enzyme analyses simplifies genome sequencing to obtain deeper coverage at particular sites, thus facilitating the identification of thousands of SNPs (single nucleotide polymorphisms) at low cost [50]–[52], as Hohenlohe et al. [53] and Amish et al. [54] demonstrated through the identification of thousands of SNPs for the accurate detection of hybrids between *Oncorhynchus mykiss* and *Oncorhynchus clarkii lewisi*.

In Brazil, most aquaculture establishments are not licensed and there is little proposed legislation regulating fish breeding [8]. In contrast, some countries have legislation that specifically addresses issues concerning hybridization: California (USA) has laws prohibiting the hybridization of fish without a proper license [2], [55]. Thus, specific laws should be implemented in Brazil to address the problems of the uncontrolled trade and management of the fish hybrids detected in several studies [12], [17], [29], [38]. Moreover, confinement measures are indispensable to avoid the widespread dissemination of fish hybrids [2], particularly physical and reproductive measures required for transgenic fish [56], [57].

Consistent with Hashimoto et al. [12], the results obtained in the present study show that genetic tools should be applied to monitor the trade of juvenile fish hybrids, representing a preventive measure for the sustainable development of the aquaculture industry, particularly because serrasalmid hybrids are fertile (*e.g.*, hybrid patinga) and hybrid tambacu can be erroneously traded as pure species. In conclusion, this multi-approach analysis (molecular, cytogenetic, and FISH methods) was useful for the detection of hybridization and the results provided new insights concerning the genome plasticity of serrasalmid species, including the occurrence of intergenus backcrossing between ♀ patinga (♀ pacu x ♂ pirapitinga) x ♂ tambaqui and the presence of an post-F1 hybrid triploid, likely derived from similar mechanisms of unreduced gametes in automixis described for other fish hybrids [42].

## References

[1] Godinho HP (2007) Estratégias reprodutivas de peixes aplicadas à aqüicultura: bases para o desenvolvimento de tecnologias de produção. Rev Bras Reprod Anim 31 3: 351–360.

[2] Hashimoto DT, Senhorini JA, Foresti F, Porto-Foresti F (2012) Interspecific fish hybrids in Brazil: management of genetic resources for sustainable use. Rev Aquaculture 4: 108–118.

[3] IBAMA (2007) Estatística da Pesca 2007: Brasil – Grandes regiões e unidades da Federação. 113 p.

[4] Porto-Foresti F, Hashimoto DT, Alves AL, Almeida RBC, Senhorini JA, et al (2008) Cytogenetic markers as diagnoses in the identification of the hybrid between Piauçu (*Leporinus macrocephalus*) and Piapara (*Leporinus elongatus*). Genet Mol Biol 31 suppl.: 195–202.

[5] Flores Nava A (2007) Aquaculture seed resources in Latin America: a regional synthesis. In: Bondad-Reantaso MG, editor. Assessment of freshwater fish seed resources for sustainable aquaculture. FAO Fisheries Technical Paper, No. 501, FAO, Rome. pp. 91–102.

[6] Honglang H (2007) Freshwater fish seed resources in China. In: Bondad-Reantaso MG, editor. Assessment of freshwater fish seed resources for sustainable aquaculture. FAO Fisheries Technical Paper, No. 501, FAO, Rome. pp. 185–199.

[7] FAO (2010) The State of World Fisheries and Aquaculture - 2010 (SOFIA). Food and Agriculture Organization of the United Nations - FAO Fisheries and Aquaculture Department, Roma.

[8] Suplicy FM (2007) Freshwater fish seed resources in Brazil. In: Bondad-Reantaso MG, editor. Assessment of freshwater fish seed resources for sustainable aquaculture. FAO Fisheries Technical Paper, No. 501, FAO, Rome. pp. 129–143.

[9] Hashimoto DT, Mendonça FF, Senhorini JA, Bortolozzi J, Oliveira C, et al (2010) Identification of hybrids between Neotropical fish *Leporinus macrocephalus* and *Leporinus elongatus* by PCR-RFLP and multiplex-PCR: tools for genetic monitoring in aquaculture. Aquaculture 298: 346–349.

[10] Prado FD, Hashimoto DT, Mendonça FF, Senhorini JA, Foresti F, et al (2011) Molecular identification of hybrids between Neotropical catfish species *Pseudoplatystoma corruscans* and *Pseudoplatystoma reticulatum* . Aquaculture Res 42: 1890–1894.

[11] Porto-Foresti F, Hashimoto DT, Prado FD, Senhorini JA, Foresti F (2013) Genetic markers for the identification of hybrids among catfish species of the family Pimelodidae. J Appl Ichthyol 29: 643–647.

[12] Hashimoto DT, Mendonça FF, Senhorini JA, Oliveira C, Foresti F, et al (2011) Molecular diagnostic methods for identifying Serrasalmid fish (Pacu, Pirapitinga, and Tambaqui) and their hybrids in the Brazilian aquaculture industry. Aquaculture 321: 49–53.

[13] Almeida-Toledo LF, Foresti F, Toledo-Filho SA, Bernardino G, Ferrari VA, et al. (1987) Cytogenetic studies in *Colossoma mitrei*, *C. macropomum* and their interspecific hybrid. In: Tiews K, editor. Selection, Hybridization and Genetic Engeneering in Aquaculture. Berlin Heenemann Verlagsgesellshaft mb II vol. 1. pp. 189–195.

[14] Nirchio M, Fenocchio AS, Swarça AC, Pérez JE, Granado A, et al (2003) Cytogenetic characterization of hybrids offspring between *Colossoma macropomum* (Cuvier, 1818) and *Piaractus brachypomus* (Cuvier, 1817) from Caicara del Orinoco, Venezuela. Caryologia 56: 405–411.

[15] Mia MY, Taggart JB, Gilmour AE, Gheyas AA, Das TK, et al (2005) Detection of hybridization between Chinese carp species (*Hypophthalmichthys molitrix* and *Aristichthys nobilis*) in hatchery broodstock in Bangladesh, using DNA microsatellite loci. Aquaculture 247: 267–273.

[16] Mair GC (2007) Genetics and breeding in seed supply for inland aquaculture. In: Bondad-Reantaso MG, editor. Assessment of freshwater fish seed resources for sustainable aquaculture. FAO Fisheries Technical Paper, No. 501, FAO, Rome. pp. 519–548.

[17] Hashimoto DT, Prado FD, Senhorini JA, Foresti F, Porto-Foresti F (2013) Detection of post-F1 fish hybrids in broodstock using molecular markers: approaches for genetic management in aquaculture. Aquaculture Res 44: 876–884.

[18] Almeida-Toledo LF, Bernardino G, Oliveira C, Foresti F, Toledo-Filho SA (1996) Gynogenetic fish produced by a backcross involving a male hybrid (female *Colossoma macropomum*×male *Piaractus mesopotamicus*) and a female *Piaractus mesopotamicus* . Boletim Técnico CEPTA 9: 31–37.

[19] Foresti F, Almeida-Toledo LF, Toledo-Filho SA (1981) Polymorphic nature of nucleolus organizer regions in fishes. Cytogenet Cell Genet 31: 137–144. 6173166 10.1159/000131639

[20] Levan A, Fredga K, Sandberg AA (1964) Nomenclature for centromeric position on chromosomes. Hereditas 52: 201–220.

[21] Yang F, O'Brien PC, Milne BS, Graphodatsky AS, Solanky N, et al (1999) A complete comparative chromosome map for the dog, red fox, and human and its integration with canine genetic maps. Genomics 62 2: 189–202. 10610712 10.1006/geno.1999.5989

[22] Pendás AM, Moran P, Freije JP, Garcia-Vazquez E (1994) Chromosomal mapping and nucleotide sequence of two tandem repeats of Atlantic salmon 5S rDNA. Cytogenet Cell Genet 67: 31–36. 8187548 10.1159/000133792

[23] Calcagnotto D, Almeida-Toledo LF, Bernardino G, Toledo-Filho SA (1999) Biochemical genetic characterization of F1 reciprocal hybrids between neotropical pacu (*Piaractus mesopotamicus*) and tambaqui (*Colossoma macropomum*) reared in Brazil. Aquaculture 174: 51–57.

[24] Gomes LC, Simões LN, Araujo-Lima CARM (2010) Tambaqui (*Colossoma macropomum*). In: Baldisserotto B, Gomes LC, editors. Espécies nativas para a piscicultura no Brasil. Universidade Federal de Santa Maria, Santa Maria. pp. 589–606.

[25] Bartley DM, Gall AE (1991) Genetic identification of native cutthroat trout (*Oncorhynchus clarki*) and introgressive hybridization with introduced rainbow trout (*O. mykiss*) in streams associated with the Alvord Basin, Oregon and Nevada. Copeia 3: 854–859.

[26] Docker MF, Dale A, Heath DD (2003) Erosion of interspecific reproductive barriers resulting from hatchery supplementation of rainbow trout sympatric with cutthroat trout. Mol Ecol 12: 3515–3521. 14629366 10.1046/j.1365-294x.2003.02000.x

[27] Na-Nakorn U, Kamonrat W, Ngamsiri T (2004) Genetic diversity of walking catfish, *Clarias macrocephalus*, in Thailand and evidence of genetic introgression from introduced farmed *C. gariepinus* . Aquaculture 240: 145–163.

[28] Angienda PO, Lee HJ, Elmer KR, Abila R, Waindi EM, et al (2011) Genetic structure and gene flow in an endangered native tilapia fish (*Oreochromis esculentus*) compared to invasive Nile tilapia (*Oreochromis niloticus*) in Yala swamp, East Africa. Conserv Genet 12: 243–255.

[29] Gomes F, Schneider H, Barros C, Sampaio D, Hashimoto DT, et al (2012) Innovative molecular approach to the identification of the tambaqui (*Colossoma macropomum*) and its hybrids, the tambacu and the tambatinga. An Acad Bras Cienc 84: 89–97. 10.1590/s0001-37652012005000025 22534749

[30] Martino G (2002) Retrocruce de hembras híbridos (F1) (*Colossoma macropomum*×*Piaractus brachypomus*) con machos de las especies parentales. In: CIVA 2002, Comunicaciones y Foros de Discusión, Espanha. pp. 688–693.

[31] Pullin RSV (1988) Tilapia Genetic Resources for Aquaculture. International Center for Living Aquatic Resources Management. Manila, Philippines.

[32] Toledo-Filho SA, Almeida-Toledo LF, Foresti F, Bernardino G, Calcagnotto D (1994) Monitoramento e conservação genética em projeto de hibridação entre pacu e tambaqui. Cadernos de Ictiogenética 2, CCS/USP, São Paulo.

[33] Orsi ML, Agostinho AA (1999) Introdução de peixes por escapes acidentais de tanques de cultivo em rios da Bacia do rio Paraná, Brasil. Rev Bras Zool 16 2: 557–560.

[34] Paiva MP, Andrade-Tubino MF, Godoy MP (2002) As represas e os peixes nativos do Rio Grande - Bacia do Paraná - Brasil. Interciência, Rio de Janeiro

[35] Allendorf FW, Leary RF, Spruell P, Wenburg JK (2001) The problems with hybrids: setting conservation guidelines. Trends Ecol Evol 16 11: 613–622.

[36] Young WP, Ostberg CO, Keim P, Thorgaard GH (2001) Genetic characterization of hybridization and introgression between anadromous rainbow trout (*Oncorhynchus mykiss irideus*) and coastal cutthroat trout (*O. clarki clarki*). Mol Ecol 10: 921–930. 11348501 10.1046/j.1365-294x.2001.01247.x

[37] Senanan W, Kapuscinski AR, Na-Nakorn U, Miller LM (2004) Genetic impacts of hybrid catfish farming (*Clarias macrocephalus*×*C. gariepinus*) on native catfish populations in central Thailand. Aquaculture 235: 167–184.

[38] Prado FD, Hashimoto DT, Senhorini JA, Foresti F, Porto-Foresti F (2012) Detection of hybrids and genetic introgression in wild stocks of two catfish species (Siluriformes: Pimelodidae): the impacts of artificial hybridisation in Brazil. Fish Res 125: 300–305.

[39] Hashimoto DT, Parise-Maltempi PP, Laudicina A, Bortolozzi J, Senhorini JA, et al (2009) Repetitive DNA probe linked to sex chromosomes in hybrids between Neotropical fish *Leporinus macrocephalus* and *Leporinus elongatus* (Characiformes, Anostomidae). Cytogenet Genome Res 124: 151–157. 19420928 10.1159/000207523

[40] Hashimoto DT, Laudicina A, Bortolozzi J, Foresti F, Porto-Foresti F (2009) Chromosomal features of nucleolar dominance in hybrids between the Neotropical fish *Leporinus macrocephalus* and *Leporinus elongatus* (Characiformes, Anostomidae). Genetica 137: 135–140. 19430915 10.1007/s10709-009-9366-y

[41] Arai K, Fujimoto T (2013) Genomic constitution and atypical reproduction in polyploid and unisexual lineages of the *Misgurnus* Loach, a Teleost Fish. Cytogenet Genome Res 140: 226–240. 23899809 10.1159/000353301

[42] Lampert KP, Lamatsch DK, Fischer P, Epplen JT, Nanda I, et al (2007) Automictic reproduction in interspecific hybrids of poeciliid fish. Curr Biol 17: 1948–1953. 17980594 10.1016/j.cub.2007.09.064

[43] Itono M, Okabayashi N, Morishima K, Fujimoto T, Yoshikawa H, et al (2007) Cytological mechanisms of gynogenesis and sperm incorporation in unreduced diploid eggs of the clonal loach, *Misgurnus anguillicaudatus* (Teleostei: Cobitidae). J Exp Zool A Ecol Genet Physiol 307: 35–50. 17094112 10.1002/jez.a.344

[44] Lamatsch DK, Stöck M (2009) Sperm-dependent parthenogenesis and hybridogenesis in teleost fishes. In: Schön I, Martens K, van Dijk P, editors. Lost sex: the evolutionary biology of parthenogenesis. Springer, Dordrecht. pp. 399–432.

[45] Shimizu Y, Shibata N, Sakaizumi M, Yamashita M (2000) Production of diploid eggs through premeiotic endomitosis in the hybrid medaka between *Oryzias latipes* and *O. curvinotus* . Zool Sci 17: 951–958.

[46] Gomelsky B (2003) Chromosome set manipulation and sex control in common carp: a review. Aquat Living Resour 16: 408–415.

[47] Martins C, Wasko AP (2004) Organization and evolution of 5S ribosomal DNA in the fish genome. Chapter X. In: Williams CR, editor. Focus on Genome Research. Nova Science Publishers, New York. pp. 289–318.

[48] Hashimoto DT, Ferguson-Smith MA, Rens W, Foresti F, Porto-Foresti F (2011) Chromosome mapping of H1 histone and 5S rRNA gene clusters in three species of *Astyanax* (Teleostei, Characiformes). Cytogenet Genome Res 134: 64–71. 21252491 10.1159/000323512

[49] Boecklen WJ, Howard DJ (1997) Genetic analysis of hybrid zones: numbers of markers and power of resolution. Ecology 78: 2611–2616.

[50] Baird NA, Etter PD, Atwood TS, Currey MC, Shiver AL, et al (2008) Rapid SNP Discovery and Genetic Mapping Using Sequenced RAD Markers. Plos One 3 10: e3376. 18852878 10.1371/journal.pone.0003376 PMC2557064

[51] Davey JW, Hohenlohe PA, Etter PD, Boone JQ, Catchen JM, et al (2011) Genome-wide genetic marker discovery and genotyping using next-generation sequencing. Nat Rev Genet 12: 499–510. 21681211 10.1038/nrg3012

[52] Wang S, Meyer E, McKay JK, Matz MV (2012) 2b-RAD: a simple and flexible method for genome-wide genotyping. Nat Methods 9: 808–810. 22609625 10.1038/nmeth.2023

[53] Hohenlohe PA, Amish SJ, Catchen JM, Allendorf FW, Luikart G (2010) Next-generation RAD sequencing identifies thousands of SNPs for assessing hybridization between rainbow and westslope cutthroat trout. Mol Ecol Res 11: 117–122. 10.1111/j.1755-0998.2010.02967.x 21429168

[54] Amish SJ, Hohenlohe PA, Painter S, Leary RF, Muhlfeld C, et al (2012) RAD sequencing yields a high success rate for westslope cutthroat and rainbow trout species-diagnostic SNP assays. Mol Ecol Resour 12 4: 653–60. 22672623 10.1111/j.1755-0998.2012.03157.x

[55] Bartley DM, Rana K, Immink AJ (2001) The use of inter-specific hybrids in aquaculture and fisheries. Rev Fish Biol Fish 10: 325–337.

[56] Mair GC, Nam YK, Solar II (2007) Risk management: reducing risk through confinement of transgenic fish. In: Kapuscinski AR, Hayes KR, Li S, Dana G, editors. Environmental risk assessment of genetically modified organisms, Volume 3: Methodologies for transgenic fish. CABI Publishing, Cambridge. pp. 209–238.

[57] Hallerman E (2008) Application of risk analysis to genetic issues in aquaculture. In: Bondad-Reantaso MG, Arthur JR, Subasinghe RP, editors. Understanding and applying risk analysis in aquaculture. FAO Fisheries and Aquaculture Technical Paper. No. 519. FAO, Rome. pp. 47–66.

